# The Impact of a Handheld Ultrasound Device in a Rheumatic Heart Disease Screening Program in Ethiopia

**DOI:** 10.24908/pocus.v8i2.16390

**Published:** 2023-11-27

**Authors:** Zachary P Kaltenborn, Anteneh Zewde, Jonathan D Kirsch, Michelle Yates, Katelyn M Tessier, Eileen Nemec, Ronald A Johannsen

**Affiliations:** 1 Departments of General Internal Medicine and Pediatrics, University of Minnesota Medical School Minneapolis , MN USA; 2 Department of General Internal Medicine, University of Minnesota Medical School Minneapolis, MN USA; 3 Soddo Christian Hospital Soddo Ethiopia; 4 Masonic Cancer Center, Division of Cardiology, University of Minnesota, Biostatistics Core Minneapolis, MN USA; 5 Children's Minnesota Minneapolis, MN USA; 6 Division of Cardiology , Hennepin Health Care Minneapolis , MN USA

**Keywords:** Rheumatic heart disease, handheld ultrasound, screening

## Abstract

**Background: **Rheumatic heart disease (RHD) affects 33 million people in low and middle income countries and is the leading cause of cardiovascular death among children and young adults. Penicillin prophylaxis prevents progression in asymptomatic disease. Efforts to expand echocardiographic screening are focusing on simplified protocols, non-physician ultrasonographers, and portable ultrasound devices, including handheld ultrasound. Recent advances support the use of single-view screening protocols. With the increasing availability and low cost of handheld devices, studies are needed to evaluate their performance in these settings. **Methods: **We conducted a retrospective study comparing the rate of screen positive ultrasounds before and after the use of a handheld ultrasound in an RHD screening program in Ethiopia. We also performed a cross-sectional device comparison in 19 at-risk school-children participating in the rheumatic heart disease screening program. **Results: **Between March of 2019 and January of 2022, 6631 children were screened for rheumatic heart disease of whom 4029 were screened after the introduction of a handheld device. Before the use of the handheld ultrasound device 291 (11.2%) children had a screen positive ultrasounds compared with 167 (4.1%) afterwards (p<0.001). We also compared non-expert to expert interpretation by device and found a significant difference in interpretation for the Lumify (p=0.025). There was a trend towards shorter jet length by color Doppler in the handheld ultrasound device for both expert and non-expert review. **Conclusions: **Our study highlights that the screen-positive rate in a RHD screening program is influenced by the device being used in the screening process.

## Background

Rheumatic heart disease (RHD) affects 33 million people in low and middle income countries and is the leading cause of cardiovascular death among children and young adults 1. RHD develops as a consequence of acute rheumatic fever which primarily affects children and will lead to RHD in 60% of cases 2. Early identification of RHD has become a cornerstone of prevention strategies and echocardiography can identify ten times the number of affected children when compared to physical exam 3. In 2012, the World Heart Federation (WHF) updated their guideline for the diagnosis of asymptomatic or latent RHD including criteria for ‘definite’ and ‘borderline’ disease 4. Using this guideline, cross-sectional echocardiographic studies have found a substantial burden of disease around the globe 5, 6, 7, 8, 9. Once identified, latent RHD appears to follow a heterogeneous course but recent evidence supports the benefit of secondary prophylaxis with penicillin 10.

While the WHF criteria serve as the gold standard for making a diagnosis of RHD, they are impractical for population-based screening programs due to time, cost, and resource availability 11. Efforts to expand echocardiographic screening are focusing on simplified protocols, non-physician ultrasonographers, and portable ultrasound devices, including handheld ultrasound 12. For example, Beaton et al. demonstrated that pediatric cardiologists employing a simplified handheld ultrasound protocol can achieve high diagnostic accuracy 13. However, shortages of echocardiographers and cardiologists in many endemic regions limit the expansion of population-based screening programs 14. Efforts to expand the screening responsibilities to include a non-physician workforce could overcome this limitation 15. Through focused training, non-physician ultrasonographers using simplified screening protocols can achieve sensitivities and specificities for detecting early RHD ranging between 74-84% and 78-90% respectively 16, 17, 18, 19. Studies evaluating single image protocols with color Doppler of the mitral and aortic valves have demonstrated sensitivity of 73-92% and specificity of 75-100% for latent RHD and screening times of 1-4 minutes 20, 21, 22, 23. More recently, an augmented single-view screening protocol was prospectively evaluated in a cohort of school-children in Timor-Leste and demonstrated a sensitivity of 100% and a specificity of 95% when completed by an expert cardiologist using standard echocardiography equipment 24. While there is increasing evidence that single view screening protocols can adequately detect latent RHD, there is no consensus on the criteria that define a positive screen 21, 24, 25. Furthermore, implementation of handheld ultrasound protocols have not been widely evaluated and the real-world sensitivity and specificity may miss numerous cases of latent RHD while still referring multiple normal children for confirmatory echocardiograms 26. Our study aims to expand what is known about the performance of handheld devices in RHD screening programs and how they impact referrals for confirmatory echocardiograms. 

## Methods

### Study Setting

In April of 2019, Soddo Christian Hospital began operating a RHD screening program following our previously described protocol 23. The screening team consists of six locally trained RHD screeners under the supervision of an onsite physician. The screeners were recruited from a wide range of hospital staff including nurses, technicians, receptionists, and sanitation personnel. These personnel were selected through an internal application process. All screeners completed a mentored training program to become proficient in executing a single parasternal long-axis view of the heart with and without color-Doppler. 

### Study Design

From April 2019 through December 2019, the screening team used a portable Sonosite M-turbo ultrasound machine. Starting in December 2019, the screening team began to use a Philips Lumify hand-held ultrasound device. Over a two-day period in November 2019, we conducted a comparison study between ultrasound devices after which time the screening team began to use the handheld device for all future school-based screenings. On both study days, all children screened were between the ages of 15-18 and from the same school. For the screening, a portable Sonosite M-turbo ultrasound machine was used with a 5-1Mhz phased array ultrasound probe. A Nyquist limit of 72 cm/s was used for color Doppler images with a frame rate of 16.667 Hz. All children determined to be positive by the screening team, as well as a random sample of children undergoing screening, were selected for a device comparison study. To minimize differences observed between devices, all children underwent repeat screening echocardiogram using the Sonosite M-turbo by a trained pediatric cardiac sonographer. This same echocardiographer then used a Philips Lumify hand-held ultrasound device using a S4-1 Mhz phased array probe. For color Doppler imaging, the Lumify device has a fixed Nyquist limit of 60 cm/s and an auto-adjusting frame rate. Two and three second video clips were stored on the Sonosite and Philips devices respectively. Images were then transferred onto encrypted flash drives and transferred onto secure hard drives for analysis. Clips contained a parasternal long-axis view of the heart with and without color Doppler. No associated demographic information was stored, and a random number was used to link the individual between devices. Where appropriate, separate images were saved for the aortic and mitral valves. Our study intervention did not alter the recommendations of the screening team and all children determined to be positive by the screening team were referred per protocol for confirmatory echocardiogram using a modified WHF criteria as the gold standard as described previously 23. This study was reviewed and approved by the Institutional Review Boards of Soddo Christian Hospital and the University of Minnesota. 

### Study Tool

All identifying information was removed from the stored video clips. Due to obvious differences in image quality and clip duration, interpretation could not be blinded by device. All images were randomly arranged for analysis so that the interpreting study investigator was not able to compare images from one individual to another. Interpretation data were collected and managed using REDCap electronic data capture tools hosted at the University of Minnesota 27. A 16-item interpretation survey was designed to capture the required elements for determining if the ultrasound was screen positive or screen negative. For the purposes of this study and within the RHD screening program, a screening ultrasound was positive if the following criteria were met: (1) A pansystolic and multicolored regurgitation at the mitral valve by color Doppler estimated at a length of more than 1.5 cm. If the regurgitation was eccentric, an estimated length of more than 1 cm was considered positive; (2) Any regurgitation at the aortic valve; (3) Any valvular abnormalities consistent with RHD. These criteria were derived from the findings of published studies and expert consensus 10, 20, 21, 23, 24. Two study investigators reviewed all ultrasounds, one an experienced cardiologist (R.J., expert) and the other an internal medicine and pediatric hospitalist (Z.K., non-expert) with experience in the use of point of care ultrasound. This design was used to capture differences in non-expert interpretation as might occur during routine school-based screenings. 

### Statistical Analysis

The primary objective of the comparison study was to determine the agreement between devices for each reader. For each reader, the agreement between devices was summarized and compared using McNemar’s test for paired samples. A secondary objective was to determine the agreement between reviewers for each device. Demographics were compared before and after the Lumify device was implemented using Wilcoxon rank sum, Chi-square of Fisher’s exact tests, when appropriate. Screen-positive rate before and after the transition to using the handheld device was summarized and compared using logistic regression. Odds ratios (OR) and 95% confidence intervals (CI) were obtained. All reported p-values are two-sided and significance level of 0.05 was used. Statistical analyses were performed using R (version 3.6.1, R Core Team) and SAS (version 9.4, SAS Institute Inc., Cary, North Carolina).

## Results

**Figure 1:**
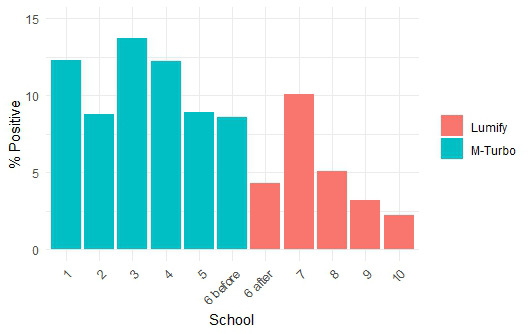
Rate of screening positivity for each school, including the school in which the transition occurred. (p<0.001).

Between April 2019 and December 2019, 2602 children ages 6-18 underwent screening for RHD (Table 1). During this time, 291 (11.2%) children had a positive screen and were referred for a confirmatory echocardiogram. Between December 2019 and January 2022, 4027 children ages 6-18 underwent screening for RHD. During this time, 167 (4.1%) had a positive screen and were referred for a confirmatory echocardiogram which was statistically significantly different than before the Lumify device was implemented (p<0.001). Logistic regression demonstrated a significantly lower odds of a positive screening exam following the introduction of the Lumify device, even after adjusting for age and gender (adjusted OR: 0.25, 95% CI: 0.2. 0.31, p<0.001, Table 2). More of the schools were classified as public following the transition (85.4% vs 19.3%, p<0.001). We also performed an analysis of the rates of screen positivity for each school over time, including the time of the transition in device which shows an abrupt change in screening positive even within the same school (p<0.001, Figure 1). Screen-positive children were referred for a confirmatory ultrasound. Complete data was not available for each confirmatory echocardiogram but there was a statistically significant change in the distribution of MR jet lengths (for those with a measurable value) as assessed by color Doppler (p< 0.001, Table 3). 

**Table 1:** Comparison of demographics and screening results for all children before and afterLumify device.

**Variable**	**All children (N=6629)**	**All children screened before Lumify device (N=2602)**	**All children screened after Lumify device (N=4027)**	P-value*
**Age**				<0.001
Mean (SD)	14.3 (2.9)	13.1 (3.1)	15.2 (2.4)	
Median (Range)	15.0 (1.0, 18.0)	13.0 (1.0, 18.0)	15.0 (7.0, 18.0)	
**Gender, n (%)**				<0.001
Number missing	2	1	1	
Female	5422 (81.8%)	2374 (91.3%)	3048 (75.7%)	
Male	1204 (18.2%)	227 (8.7%)	977 (24.3%)	
Other	1 (0.0%)	0 (0.0%)	1 (0.0%)	
**Echo screening result, n (%)**				<0.001
Negative	6171 (93.1%)	2311 (88.8%)	3860 (95.9%)	
Positive	458 (6.9%)	291 (11.2%)	167 (4.1%)	
**Confirmatory echo, n (%)**				0.110
Number missing	6202	2317	3885	
Abnormal	425 (99.5%)	285 (100.0%)	140 (98.6%)	
Normal	2 (0.5%)	0 (0.0%)	2 (1.4%)	
**MR jet length, n (%)**				<0.001
Number missing	6204	2317	3887	
1-1.4 cm	130 (30.6%)	51 (17.9%)	79 (56.4%)	
1.5-2 cm	287 (67.5%)	229 (80.4%)	58 (41.4%)	
>2 cm	8 (1.9%)	5 (1.8%)	3 (2.1%)	
**School, n (%)**				-
1	335 (5.1%)	0 (0.0%)	335 (8.3%)	
2	600 (9.1%)	600 (23.1%)	0 (0.0%)	
3	508 (7.7%)	255 (9.8%)	253 (6.3%)	
4	501 (7.6%)	501 (19.3%)	0 (0.0%)	
5	502 (7.6%)	502 (19.3%)	0 (0.0%)	
6	247 (3.7%)	247 (9.5%)	0 (0.0%)	
7	1285 (19.4%)	0 (0.0%)	1285 (31.9%)	
8	497 (7.5%)	497 (19.1%)	0 (0.0%)	
9	897 (13.5%)	0 (0.0%)	897 (22.3%)	
10	1257 (19.0%)	0 (0.0%)	1257 (31.2%)	
**School location, n (%)**				-
Urban	6629 (100.0%)	2602 (100.0%)	4027 (100.0%)	
**School type, n (%)**				<0.001
Private	2688 (40.5%)	2100 (80.7%)	588 (14.6%)	
Public	3941 (59.5%)	502 (19.3%)	3439 (85.4%)	
*P-value is for Wilcoxon rank-sum test for age and Chi-square or Fisher’s exact test for categorical variables.				

**Table 2:** Logistic regression results for echo screening result for all children before and afterLumify device.

-	**Unadjusted**		**Adjusted**	
	**OR (95% CI)**	P-value*	**OR (95% CI)**	P-value*
**Before Lumify device**	Reference	<0.001	Reference	<0.001
**After Lumify device**	0.34 (0.28, 0.42)		0.25 (0.20, 0.31)	
*Logistic regression models with and without adjustment for age and gender were used.				

**Table 3:** Summary of MR jet lengths as determined by color Doppler for those children who underwent confirmatory echocardiogram following device transition.

**Variable**	**MR jet lengths before Lumify transition (N=291)**	**MR jet lengths after Lumify transition (N=167)**	P-Value*
MR jet length, n (%)			<0.001
Did not receive confirmatory echocardiogram	6	25	
1-1.4 cm	51 (17.9%)	79 (55.6%)	
1.5-2 cm	229 (80.4%)	60 (42.3%)	
>2 cm	5 (1.8%)	3 (2.1%)	
*P-value is for Fisher’s exact test.			

During the two-day in-field comparison, 202 children underwent screening ultrasounds. A total of 13 children had screen positive exams. An additional 5 children were randomly selected to have an ultrasound with both devices for a total of 19 children were included in the device comparison. All study ultrasounds contained adequate visualization of the mitral valve to assess for soft tissue and color Doppler abnormalities. All ultrasounds contained adequate imaging of the aortic valve. Four ultrasounds (3 M-turbo and 1 Lumify) contained inadequate color Doppler of the aortic valve by expert review secondary to imperfect capture of the aortic valve by the Doppler window. 

**Figure 2:**
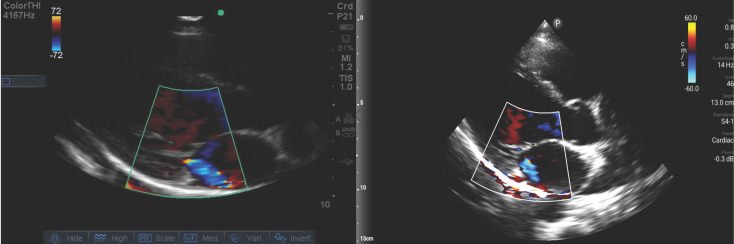
Side-by-side images from the same child illustrating the differential appearance of mitral regurgitation by color Doppler between the M-turbo (A) and the Lumify (B). These images represent the most abnormal jet from the respective video clips as determined by expert review.

When comparing non-expert to expert interpretation by device, the Lumify was more likely to be interpreted as screen negative (p=0.025, Table 4). We undertook a detailed evaluation of the three children in which non-expert interpretation was in agreement with the M-turbo but not the Lumify (Table 5). There were important differences between expert and non-expert interpretation of the color Doppler images which impacted the screen status. Review of the observed mitral regurgitation in these children demonstrated more subtle color Doppler findings when viewed using the Lumify device (Figure 2). 

**Table 4:** Comparison of screen status by reviewer for each device.

**M-Turbo**			
-	**Non-expert review positive**	**Non-expert review negative**	P-value*
**Expert review positive**	5 (26.3%)	1 (5.3%)	0.317
**Expert review negative**	0 (0.0%)	13 (68.4%)	
**Lumify**			
-	**Non-expert review positive**	**Non-expert review negative**	P-value*
**Expert review positive**	2 (10.5%)	5 (26.3%)	0.025
**Expert review negative**	0 (0.0%)	12 (63.2%)	
*P-value is for McNemar’s test for paired samples.			

**Table 5:** Comparison of the mitral valve regurgitation in three children where there was disagreement in screen status between expert and non-expert review.

-	**Expert**		**Non-expert**	
Device	M-Turbo	Lumify **	M-turbo	Lumify
**Child 1**				
Mitral jet length	1-1.5 cm	1-1.5 cm	1.5-2 cm	1.5-2 cm
Pansystolic	Yes	Yes	Yes	No
Eccentric	Yes	Yes	Yes	No
Multi-colored	No	Yes	Yes	Yes
Screen Positive	Yes	Yes	Yes	No
**Child 2**				
Mitral jet length	1.5-2 cm	1-1.5 cm	> 2 cm	< 1 cm
Pansystolic	Yes	Yes	Yes	No
Eccentric	Yes	Yes	Yes	No
Multicolored	Yes	Yes	Yes	No
Screen Positive	Yes	Yes	Yes	No
Child 3*				
Mitral jet length	1-1.5 cm	1-1.5 cm	1.5-2 cm	1-1.5 cm
Pansystolic	Yes	Yes	Yes	No
Eccentric	Yes	No	Yes	No
Multicolored	Yes	No	Yes	No
Screen positive	Yes	Yes	Yes	No
* Child 3: On Lumify device, thickening was noted on the mitral valve but not seen on corresponding M-turbo image for expert review. ** Lumify: Color Doppler Frame rates varied per child: 1. 17 Hz., 2. 14 Hz., 3. 15 Hz.				

## Discussion

Introduction of a handheld ultrasound device into an active school-based RHD screening program in Ethiopia significantly decreased the number of screen-positive ultrasounds. Among a small-sample of adolescent children, our device comparison highlights important and measurable differences between the handheld Philips Lumify and the Sonosite M-Turbo. When comparing screen status by device, there was a statistically significant difference between expert and non-expert interpretation using the Lumify. Overall, mitral jets trended towards appearing shorter when viewed on the Lumify. 

Single-view screening protocols for the detection of latent RHD are increasingly demonstrating strong sensitivity and specificity 21, 24. However, most studies to date have not evaluated the impact of handheld devices in the field. With the increasing availability and low cost of these devices, teams around the world have already started to employ modified versions of published single-view screening criteria 6. While these efforts are needed to help realize the potential of population based screening for RHD, our study demonstrates that there are important differences between ultrasound devices that may impact the rate of screen-positivity even when the screening protocol remains constant. 

## Limitations

Our study has many important limitations. While all images were captured by the same experienced pediatric cardiac sonographer, differences in image acquisition may explain some of the differences observed. Some differences are expected between expert and non-expert interpretation of echocardiograms for RHD screening 16, 18, 28, 29. However, it is notable that agreement regarding screen status on the M-turbo device was extremely high between reviewers with no case of screen-positive valvular regurgitation being missed by non-expert review suggesting that differences in appearance between the ultrasound devices explains the discordance in screen status. However, the careful and nuanced review as performed by study personnel may not accurately capture the more rapid in-field interpretation done by the screening team which may favor referral, even for cases where the screening test does not meet rigorous criteria. Given the importance of mitral regurgitation in the early detection of RHD, we believe this trend is largely responsible for the observed change in screen-positive ultrasounds. 

There are inherent differences between the devices. The Phillips Lumify has a fixed Nyquist limit of 60 cm/s whereas the Sonosite M-turbo can be adjusted. For the purposes of this study, the Nyquist limit on the M-turbo was set a 72 cm/s. The Nyquist limit on the M-turbo was not adjusted to minimize interruptions to the standard imaging settings already employed by the screening team. Interestingly, our observed trend towards lower jet lengths in the Lumify are not readily explained by the lower Nyquist limit as one would expect increased aliasing and artifact given the high velocities being observed 30. Reducing the Nyquist limit in the M-turbo would have exacerbated the observed discrepancies in jet length which suggest the jet length discrepancy is related to other factors. 

Most importantly, we report primarily on the impact of the handheld device on the rate of screen-positivity from the schools. As described, all children with a screen-positive exam were referred for a confirmatory echocardiogram using a modified version of the WHF criteria. Complete confirmatory results were not available for all children but we did observe a significant change in the distribution of MR as estimated by color Doppler. Our team has since developed a more complete and accurate process of capturing the complete results of the confirmatory echocardiograms. However, the lower number of referrals with a smaller number of intermediate jet lengths does imply a higher degree of specificity. How the Lumify device may have impacted sensitivity and this important question is not well answered by this study. Rigorous studies evaluating non-expert ultra sonographers typically show a high-degree of sensitivity with most limitations found in the specificity. However, recent work by others in the field using a similar handheld device have also found that programs may need to consider impacts that are device specific 31. Our observed change in screen-positivity appeared to persist even after controlling for important confounding variables such as age and gender. Ongoing quality improvement monitoring within our program is notable for extremely rare falsely negative screening echocardiograms with increasing specificity noted overtime which is an important source of confounding. However, when analyzed by school, there was an abrupt change in the screen-positive rate. Sequential improvement in screener sensitivity would likely have been observed to occur over a longer and more gradual period of time. It is important to note that the distribution of public versus private schools varied across the time of device transition and may have impacted the rate of screen-positivity we observed. Our program also started in the center of town for ease of access by car. We then sequentially worked our way to the outskirts of town and then into the surrounding areas. The definitions of urban versus rural are challenging to define in this environment. Though all schools presented in this study were categorized as urban, many were located on the outskirts of town and still required rough travel by dirt roads and off-road vehicle. 

## Conclusion

The differences observed in our study suggest that the thresholds used to determine the ideal positive screen may be influenced by unique and unpredictable performance characteristics of the ultrasound device being utilized. Our study highlights that the ideal criteria for determining a screen positive ultrasound may vary by ultrasound device as well as screener experience and skill. Criteria that move away from length based estimations of color Doppler jets and focus on other characteristics of pathological regurgitation seen in latent RHD may be preferable 24. Even among expert cardiologists, length based estimations of mitral and aortic regurgitation are subject to variability when making a confirmatory diagnosis 32. Until further evidence is available, the use of handheld devices for RHD screening should be approached cautiously to avoid missing cases that would otherwise be referred for confirmatory echocardiograms. 

## Disclosures

Zachary P. Kaltenborn received Funding for this project from the University of Minnesota Center for Global Health and Social Responsibility Faculty Travel Awards Program 

Jonathan D Kirsch obtained the Philips Lumify used in this study through the University of Minnesota Foundation, Faculty Research and Equipment Grant, 2018

Research reported in this publication was supported by NIH grant P30 CA77598 utilizing the Biostatistics Core shared resource of the Masonic Cancer Center, University of Minnesota and by the National Center for Advancing Translational Sciences of the National Institutes of Health Award Number UL1TR002494. The content is solely the responsibility of the authors and does not necessarily represent the official views of the National Institutes of Health.

## Conflicts of Interest

All authors have reviewed the above manuscript and attest that they have no financial or non-financial conflicts of interest. 

## Ethical Approval and Consent to Participate

This study was reviewed and approved by the Institutional Review Board at the University of Minnesota and determined to be a non-human research subjects study (STUDY00008308). The IRB determined that the proposed activity is not research involving human subjects as defined by DHHS and FDA regulations. The RHD screening program is done in cooperation with local educational and health ministries. The consent process for participation in the screening program is described above and was not altered by this study. 

## Availability of data and materials

All data generated or analyzed during this study are included in this published article and its supplementary information files.
